# Web Tool for Navigating and Plotting SomaLogic ADAT Files

**DOI:** 10.5334/jors.166

**Published:** 2017-09-08

**Authors:** Foo Cheung, Giovanna Fantoni, Maria Conner, Brian A Sellers, Yuri Kotliarov, Julián Candia, Katherine Stagliano, Angélique Biancotto

**Affiliations:** Trans-NIH Center for Human Immunology, Autoimmunity and Inflammation, National Institutes of Health, Bethesda, MD 20892, US

**Keywords:** ADAT, Shiny, SOMAscan, proteomic

## Abstract

SOMAscan™ is a complex proteomic platform created by SomaLogic. Experimental data resulting from the assay is provided by SomaLogic in a proprietary text-based format called ADAT. This manuscript describes a user-friendly point and click open source, platform-independent software tool designed to be used for navigating and plotting data from an ADAT file. This tool was used either alone or in conjunction with other tools as a first pass analysis of the data on several different on-going research projects. We have seen a need from our experience for a web interface to the ADAT file so that users can navigate, generate plots, perform QC and conduct statistical analysis on their own data in a point and click manner. After several rounds of interacting with biologists and their requirements with respect to data analysis, we present an online interactive Shiny Web Tool for Navigating and Plotting data contained within the ADAT file. Extensive video tutorials, example data, the tool and the source code are available online.

## (1) Overview

### Introduction

SomaLogic’s proteomic platform called SOMAscan™ [1] can measure more than 1300 proteins from biological fluids. At the National Institutes of Health (NIH), The Center for Human Immunology, Autoimmunity and Inflammation (CHI) has earned certification as a deployment center for the SOMAscan™ assay. The SOMAscan assay is a highly multiplexed proteomic platform that uses SOMAmer (Slow Off-rate Modified Aptamers) reagents to selectively bind and quantify proteins in fluid matrices. Relative protein concentrations are converted to measurable nucleic acid signals that are quantified by hybridisation to DNA microarrays. The assay results are provided by SomaLogic in a proprietary text-based format called ADAT, containing the reagent intensities, sample and sequence annotation and experimental metadata. Currently, those who would like to perform data analysis on their own data set derived from the SomaLogic’s SOMAscan™ assay platform have several options including the following: 1) SomaSuite [2] 2) using a R package eg: readat [3] or 3) writing their own computer program. Although Option 1 is suitable for many users as SomaSuite is a windows based software, it is not open source. Option 2 would be an option to many bioinformaticians but it does require knowledge of R which can be both difficult and time consuming to some users. Option 3, is time prohibitive as it will require learning the ADAT specification and subsequent software development from the ground up. Although the ADAT file is in a text tab-delimited format it does not conform to a common format. One would need to understand the ADAT structure and how to get data in or out of them.

We created a tool that can rapidly communicate, share ideas and generate some useful information directly from the uploaded ADAT file, including heatmaps, box and line plots, Principle Component Analysis (PCA) and other statistical data.

Results are conveyed through interactive plots on the web and can quickly draw attention to the users and collaborators of key results. Interactive plots make it possible to uncover hidden patterns that could drive further hypothesis that would not be apparent from looking at statistics alone.

### Implementation and architecture

This web application is written using the shiny framework [4], a package from RStudio that can be used to build interactive web pages with R [5]. The code is split into two parts, the user interface and the server-side containing the logic. The HTML widgets framework [6] was used for binding JavaScript data visualizations in R and numerous HTML widgets and R packages are used including plotly [7], DT [8], d3heatmap [9], shinydashboard [10] and ggplot2 [11].

The user interface is currently organized into the following dashboard tabs: “Upload ADAT file”, “BoxPlots”, “Heatmap And Statistics”, “PCA”, “Download” and “Help”, which will be discussed in the following sections.

“Help” buttons are provided on each page so that the user can be familiar with available features. We focused on providing an easy to use interface with ability to generate informative charts that has a professional look and feel. Creating plots (Box, Heat maps or PCA plots) is just a matter of loading in a ADAT file, customizing the layout through the “Options” box and if necessary filtering samples through the “Filter Samples” box. The “Options” box allows the user to manipulate ranges, change input parameters and the data at the same time.

### Illustrated Examples

**Figure 1:**
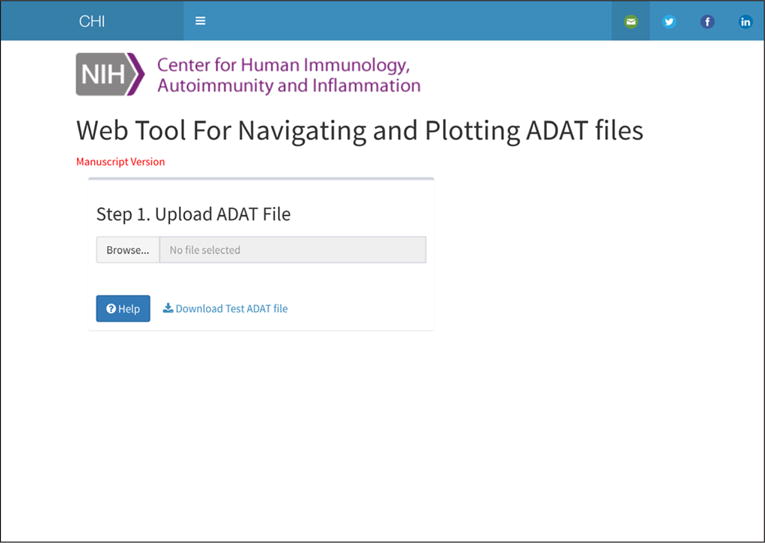
Upload ADAT page.

Documentation, in the form of videos showing how to perform the following tasks, is available from the application itself https://foocheung.shinyapps.io/adat/. To demonstrate the features of the application, we used a dataset representing plasma samples from 20 US adults aged between 35 and 75 years old [2]. The first step requires the user to upload the SOMAscan™ ADAT file. Launching the application first loads the “Upload .adat File” dialog that allows the user to upload their ADAT file. The sidebar is disabled and all tabs are hidden until an ADAT file is loaded (Figure 1). (Note the side bar menu can be made visible even without ADAT file, when clicking on a small icon on top of the page).

**Figure 2:**
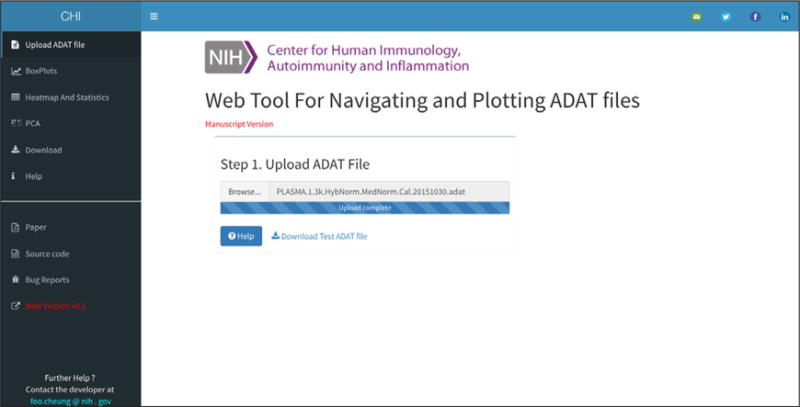
ADAT tab after the upload of an ADAT file.

Once an ADAT file has been uploaded, Sidebar menu items and tabs are revealed allowing the user to select any of the six available tabs (“Upload .adat file”, “BoxPlots”, “Heatmap And Statistics”, “PCA”, “Download” and “Help”) as shown on Figure 2.

**Figure 3:**
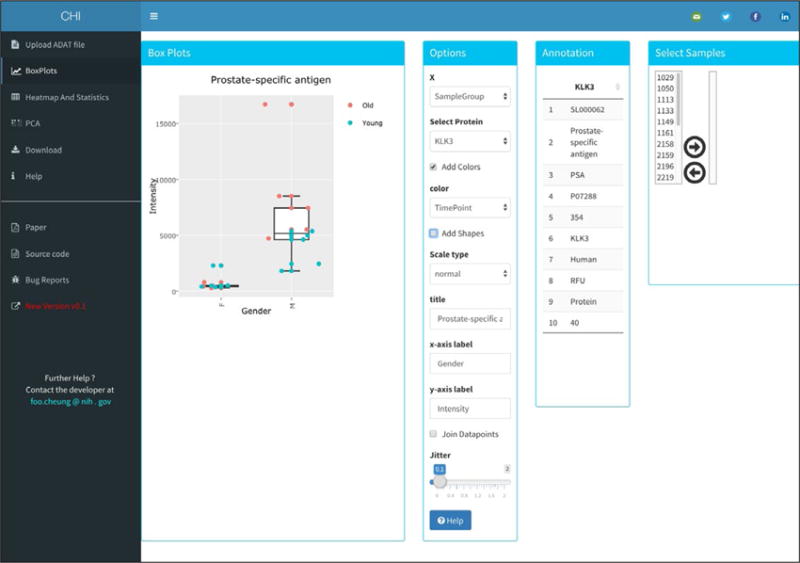
BoxPlots tab page, an example output.

The “BoxPlots” page contains four boxes in a row-based layout. The first box going from left to right) labeled “Box Plots” allows the user to display interactive plots for selected protein as shown on Figure 3.

Multiple options are currently available for customizing the interactive and downloadable plots. The second box labeled “Options” provides the user multiple options for box plot customization. The user can select different variables to be plotted on X (categories) and Y (protein intensities) axis. Different shapes and colors can be used to represent samples. The user can add a main title or axis labels to the plot, by entering them on the correspondent text box.

The third box labeled “Annotation” displays a table that contains proteins annotation from the ADAT file such as: SomaId, TargetFullName, Target, UniProt, EntrezGeneID, EntrezGeneSymbol, Organism, Units, Type and Dilution. Finally, the fourth panel labelled “Select Samples” allows the user to filter individual samples by using a dueling select box: once activated, samples in the left box will be immediately removed from the plot.

**Figure 4:**
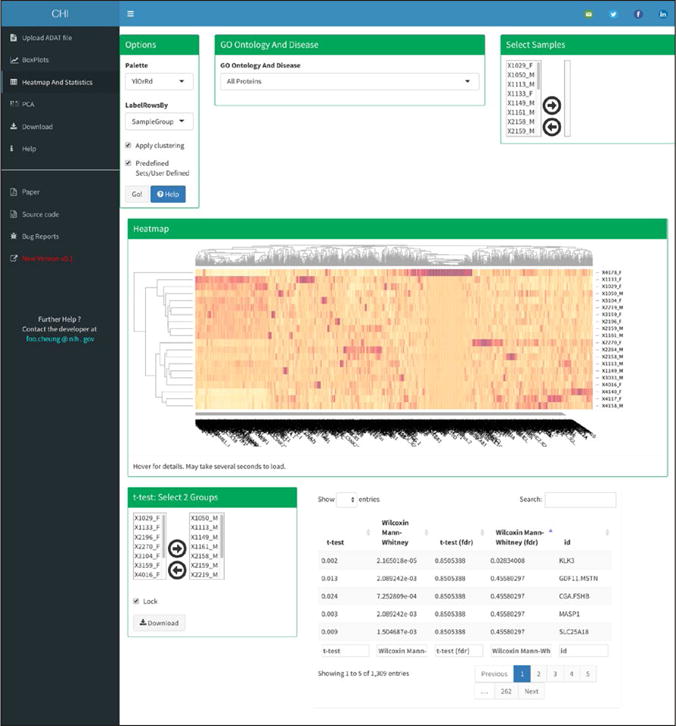
Heatmap and Statistics tab, an example output.

The ‘Heatmap And Statistics’ (Figure 4) tab shows five boxes:

“Options”, “GO Ontology And Disease” or “Select Proteins”, “Select Samples”, “Heatmap” and “t-test: Select 2 Groups”. The first box entitled “Options” allows the user to modify various options for heatmap plot. Drop down boxes are available to change the heatmap color gradients and labels while check boxes allow the user to toggle whether to cluster or not the heatmap’s rows and columns and to select subsets of proteins based on GO process and Disease ID or generate a user defined set. The GO and Disease annotation was kindly provided by SomaLogic (Version 3). Clicking on the “Go” button generates a new heatmap plot. The third box labeled “Select Samples” allows the user to filter samples. The fourth box displays the actual interactive heatmap that allows the user to get the row/column/value under the mouse cursor and zoom in by dragging a rectangle over the heatmap. The fifth allows the user to select two groups of samples and will automatically execute a t-test and Mann Whitney test comparing these groups. The output of raw and FDR corrected p-values is displayed as an interactive data table that can be searched, filtered, and sorted as required.

**Figure 5:**
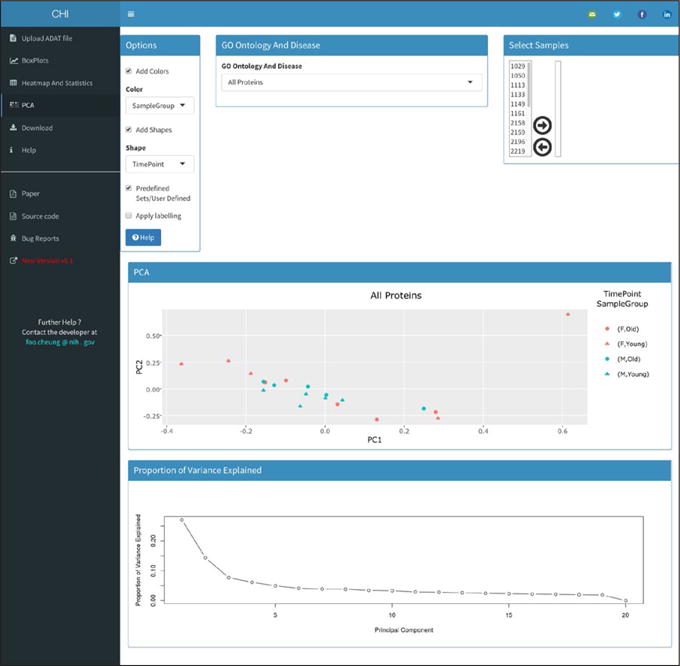
PCA tab, an example output.

The “PCA” tab allows users to run Principal Component Analysis which has become a standard procedure for looking at population substructure to identify co-founders such as batch effects, and to identify relatedness between observations. As for previous tabs, there is an “Options” box allows the user to control the output of the plot, a “Select Samples” box panel to filter the samples and a box labelled “GO Ontology And Disease” that allows the user to select proteins associated with a GO process and Disease ID or generate an user defined set (Figure 5).

To export the data in a CSV file, visit the “Download” tab where the user can download the complete data or a sub-set based on GO process and Disease ids. The Help tab allows the user to view tutorial videos.

### Quality control

The software has been through several rounds of QC by manually checking plots and statistics, comparing the output with commercially available third party tools and in-house developed pipelines. Another method for quality control is user feedback, this web tool is supported by feedback, bug reports and feature wishes from numerous users. The application has been tested in modern web browsers, including Google Chrome, Safari, Firefox, and IE10+ and in several operating systems, including Windows, Mac, Linux and Chrome.

## (2) Availability

The web application is available at https://foocheung.shinyapps.io/adat/ and the source code is available at https://github.com/foocheung/adat.

### Operating system

A platform-independent software package, compatible with modern web browsers (IE10+, Google Chrome, Firefox, Safari, etc.).

### Programming language

R, JavaScript.

### Additional system requirements

None.

### Dependencies

Imports a number of R packages (see code for most up-to-date list).

### Installation

We expect that most users will use the web tool directly from the website https://foocheung.shinyapps.io/adat/ but users can also install this web tool from source code https://github.com/foocheung/adat if required.

### List of contributors

Please list anyone who helped to create the software (who may also not be an author of this paper), including their roles and affiliations.

### Software location

#### Code repository

***Name:*** GitHub
***Identifier:*** https://github.com/foocheung/adat
***Licence:*** Apache
***Date published:*** 01/03/17

#### Language

English.

## (3) Reuse potential

As a standalone program, this web tool provides researchers a way to analyze their own data in an intuitive, ‘clickable’ manner. Currently the tool generates boxplots, heatmaps, PCA plots and provides statistical comparisons between two groups of samples. This program has a high reuse potential “as is” and has already been a useful tool for several researchers working on different projects from a wide spectrum of biology regardless of coding experience. Future development will be driven by user requirements and needs.
